# Proteome dataset of peripheral blood mononuclear cells in postpartum dairy cows supplemented with different sources of omega-3 fatty acids

**DOI:** 10.1016/j.dib.2021.107785

**Published:** 2022-01-04

**Authors:** Gitit Kra, Nataly Nemes-Navon, Jayasimha Rayalu Daddam, Lilya Livshits, Shamay Jacoby, Yishai Levin, Maya Zachut, Uzi Moallem

**Affiliations:** a Department of Ruminant Science, Institute of Animal Sciences, Agricultural Research Organization, Volcani Center, Israel; b Department of Animal Science, The Robert H. Smith Faculty of Agriculture, Food and Environment, The Hebrew University of Jerusalem, Rehovot 76100, Israel; c The Nancy and Stephen Grand Israel National Center for Personalized Medicine, Weizmann Institute of Science, Rehovot 7610001, Israel

**Keywords:** Dairy cows, Peripheral blood mononuclear cells, Proteomic analysis, Omega-3 fatty acids

## Abstract

This article contains raw and processed data related to research published by Kra et al. [1]. There is a scarce knowledge on the proteome of peripheral blood mononuclear cells (PBMC) during the transition period in dairy cows. In human research, proteomics PBMC is used in order to gain insight into inflammatory diseases and syndromes. Dietary fats, and specifically omega-3 (n-3) FA, can moderate the immune fluctuation caused by parturition through improvements of the immune function [2]. Therefore, this study aim was to characterize the changes that may occur in proteome of PBMC during transition, as influenced by different n-3 FA supplementation. Proteomics data of PBMC was obtained from postpartum dairy cows supplemented peripartum with either encapsulated saturated fat (CTL), encapsulated flaxseed oil that is enriched with ALA (α-linolenic acid; FLX) or encapsulated fish oil that is enriched with EPA (eicosapentaenoic acid) and DHA (docosahexaenoic acid; FO).The analysis was done by liquid chromatography-mass spectrometry from PBMCs protein extraction. The cells were collected from six cows per treatment during the 1^st^ week postpartum. Quantification of differential abundance between groups was done using MS1 intensity based label-free. Label-free quantitative shotgun proteomics was used for characterization. This novel dataset of proteomics data from PBMC contains 3807 proteins; 44, 42 and 65 were differently abundant (*P* ≤ 0.05 and FC ± 1.5), in FLX vs. CTL, FO vs. CTL and FLX vs. FO, respectively; these findings are discussed in our recent research article (Kra et al., 2021). The present dataset of PBMC proteome adds new information regarding the effects of n-3 FA on the immune system, while providing reference for PBMC proteome in postpartum dairy cows.

## Specifications Table

**Table:** 

Subject	Animal Science
Specific subject area	Physiology and nutrition of ruminants
Type of data	Table
How data was acquired	Liquid Chromatography-Mass spectrometry: nanoESI+Q Exactive HF
Data format	Raw, identifications, analyzed
Parameters for data collection	Forty-two multiparous dairy cows were divided into 3 groups at 257 days of pregnancy that were fed a basal diet and supplemented until 60 days in lactation either with: (i) CTL – (n = 14) encapsulated saturated fat; (ii) FLX (n = 14) – encapsulated flaxseed oil enriched with ALA; and (iii) FO – (n = 14) encapsulated fish oil enriched with EPA and DHA. Blood sample was taken into a heparin-coated tube on day 3–12 (average 7 days) PP from 24 cows (8 cows from each treatment) for PBMC separation as previously described (Mann et al., 2018).
Description of data collection	PBMC was selected randomly from 18 cows (6 from each group) for proteomic analysis. PBMC were analyzed by Liquid Chromatography-Mass spectrometry following protein extraction. Differential abundance was quantified using MS1 intensity based label-free.
Data source location	• Institution: ARO Volcani Center • City/Town/Region: Rishon Lezion • Country: Israel • Latitude and longitude for collected samples: latitude: 31.989347, longitude:34.820444
Data accessibility	With the article. Data are available via ProteomeXchange with identifier PXD029683. Project Webpage: http://www.ebi.ac.uk/pride/archive/projects/PXD029683 FTP Download: ftp://ftp.pride.ebi.ac.uk/pride/data/archive/2021/11/PXD029683
Related research article	G. Kra, N. Nemes-Navon, J. R. Daddam, S Jacoby, Y. Levin, M. Zachut, U. Moallem, Proteomic analysis of peripheral blood mononuclear cells and immune function in postpartum dairy cows supplemented with different sources of omega-3 fatty acids. J. of Proteomics 2021, 246:104313. doi: 10.1016/j.jprot.2021.104313.

## Value of the Data

• This work provides the first documentation of proteome dataset from PBMC of postpartum (week 1) dairy cows that were supplemented peripartum with different n-3 FA, quantifying 3807 proteins.
• The proteome dataset from bovine PBMC can be used as either biological markers for PP immune function or inflammatory markers as affected by dietary supplementation of n-3 FA in postpartum dairy cattle.
• We examined the different abundance among all groups: FLX vs. CTL, FO vs. CTL and FLX vs. FO, and found that 44 proteins were differently abundant (*P* ≤ 0.05 and FC ± 1.5) between FLX and CTL, 42 between FO and CTL, and 65 between CTL and FLX vs. FO cows.
• In FLX vs. CTL, the abundance of the p65 subunit of transcription factor NF-κB was higher, whereas albumin, C4b-binding protein and complement factor H levels were lower. In FLX vs. FO, complement factors B and H and hemopexin were higher. Further research can be done to explore the characteristics and functioning of these proteins in the inflammatory and immune function as affected by dietary supplementation to peripartum dairy cows.

## 1 Data Description

This data describes the proteome of PBMC at week 1 postpartum in dairy cows supplemented peripartum with different n-3 FA. Supplementary Table 1 contains the dataset of 3807 identified and quantified proteins obtained by proteomic analysis, as well as statistical analysis of differentially abundant proteins among all groups (*P* ≤ 0.05 and FC ± 1.5). The full list of peptides is presented in Supplementary Table 2.

## 2 Materials and Methods

### 2.1 Animals and procedures

The study experimental protocol was approved by the Volcani Center Animal Care Committee (approval number IL 797/18), along with the relevant guidelines and regulations. The experiment was performed at the experimental dairy farm of Volcani Center in Rishon LeZion, Israel. The data presented in this paper are part of a larger study examining the effects of different n-3 FA supplemented peripartum on production, inflammatory and immune parameters, subpopulation of white blood cells, and proteome of PBMC. Full details on animal management and handling are provided in the companion paper [1].

Forty-two multiparous dry dairy cows were assigned into 3 groups at 257 days of pregnancy and supplemented up to 60 days postpartum a basal diet supplemented either with: (i) CTL – (n = 14) encapsulated saturated fat; (ii) FLX (n = 14) – encapsulated flaxseed oil that is enriched with ALA; and (iii) FO – (n = 14) encapsulated fish oil that is enriched with EPA and DHA. Blood sample were collected using a heparin-coated tube on day 3–12 (average 7 days) PP from 24 cows (8 cows from each treatment) for PBMC. Details on cows and procedures are in Kra et al. [1].

### 2.2 PBMC preparation

The PBMC blood sample was taken into a heparin-coated tube on day 3–12 (average 7 days) PP from 24 cows (8 cows from each treatment). The PBMC separation was done as previously described [1], [3] with some modifications. Briefly, the whole blood was diluted (at a 1:1 ratio) using PBS with phosphatase and protease inhibitors (10 mM sodium fluoride, S7920; 1 mM sodium orthovonadate, S6508; and 10 mM sodium β-glycerophosphate, BGP-G5422, all from Sigma-Aldrich, Israel and dissolved in PBS). The diluted blood was gently layered on a ficoll layer (10771, Histopaque 1077, Sigma-Aldrich, Israel). The PBMC were collected into a fresh tube following a centrifugation for 30 min at 1400g 4 °C in slow release and acceleration mode. The PBMC were then washed with cold PBS. Finally, the cells were reconstituted in PBS containing protease and phosphatase inhibitors (P5726, phosphatase inhibitor cocktail; P8340, protease inhibitor cocktail, 1% v/v each, Sigma-Aldrich, St. Louis, MO). Samples were frozen at −80 °C at a concentration of 1 × 107 cells/mL pending protein extraction for proteomics.

### 2.3 Sample preparation for proteomic analysis

We randomly selected PBMC from 18 cows (6 from each treatment) for the proteomic analysis. The samples were thawed and sediment by centrifuged for 15 min at 500g, 4 °C. The supernatant was discarded and 0.5-mm glass beads (11079105, BioSpec, Bartlesville, OK) were added to the bottom of the vial. The cells were then ground in a homogenizer (BeadBug, Benchmark Scientific, Sayreville, NJ) with 1 mL lysis buffer [5% (w/v) SDS in 100 mM Tris-HCl buffer containing 1% (v/v) PMSF, P7626; 1% phosphatase inhibitor, P5726; and 1% protease inhibitor, P8340, all from Sigma-Aldrich, St. Louis, MO]. Following a centrifugation for 15 min at 20,000g, 4 °C, the protein concentration of the sample was analyzed by bicinchoninic acid (BCA) standard assay (9470BCAstand, Cyanagen, Bologna, Italy), then snap-frozen and stored at −80 °C. The proteomics process was achieved by incubation at 96 °C for 5 min, followed by six 30-s cycles of sonication (Bioruptor Pico, Diagenode, Denville, NJ). Proteins were reduced using 5 mM DTT and alkylated with 10 mM iodoacetamide under cover. Each sample was loaded onto S-Trap microcolumns (ProtiFi, Huntington, NY) according to the manufacturer's instructions. In short, the samples were washed with methanol containing 50 mM ammonium bicarbonate, 90:10 (v/v). The samples were then processed with trypsin for 1.5 h at 47 °C. The digested peptides were eluted using 50 mM ammonium bicarbonate; trypsin was added to this fraction and incubated overnight at 37 °C. Two more elusions were performed using 0.2% (v/v) formic acid and 0.2% formic acid in 50% (v/v) acetonitrile (ACN). The pull of all three elusions was vacuum-centrifuged to dry. Samples were kept at −80 °C until analysis.

### 2.4 LC/MS

All chromatographic steps were done using ULC/MS-grade solvents. Sample was loaded for split-less Nano-Ultra Performance Liquid Chromatography (UPLC) (10 kpsi nanoAcquity; Waters, Milford, MA). The mobile phase consisted of: (A) H2O + 0.1% formic acid and (B) ACN + 0.1% formic acid. RP Symmetry C18 trapping column (180 µm id, 20 mm length, 5 µm particle size; Waters) was used to desalt the samples online. The peptides were then separated using a T3 HSS nano-column (75 µm id, 250 mm length, 1.8 µm particle size; Waters) at 0.35 µL/min. The following gradient was used to elute the peptide from the column into the mass spectrometer: 4% to 27% B in 155 min, 27% to 90% B in 5 min, maintained at 90% B for 5 min and then back to initial conditions.

The Nano-UPLC was coupled online through a NanoESI emitter (10 µm tip; New Objective, Woburn, MA) to a Q Exactive HF mass spectrometer (Thermo Scientific). Data were acquired in data-dependent acquisition mode using the Top10 method. MS1 resolution was set to 120,000 (at 200m/z), mass range of 375–1650 m/z, automatic gain control (AGC) of 3e6 and maximum injection time was set to 60ms. MS2 was performed by isolation with the quadrupole, width of 1.7Th, 27 NCE, 15k resolution, AGC target of 60ms and dynamic exclusion of 45s.

### 2.5 Proteomics data analysis

Raw data were processed with MaxQuant v1.6.6.0. The Andromeda search engine was used for searching against the bovine sequences from UniprotKB, and appended with common laboratory protein contaminants. The trypsin enzyme specificity was set with up to two missed cleavages allowed. Fixed modification was set to carbamidomethylation of cysteines and variable modifications were set to oxidation of methionines, and deamidation of N or Q. Peptide precursor ions were searched with a maximum mass deviation of 4.5 ppm and fragment ions with a maximum mass deviation of 20 ppm. Peptide and protein identifications were filtered at a false discovery rate of 1% using the decoy database strategy, while minimal peptide length was 7 amino acids. The match-between-runs option was used for peptide identifications across samples. Searches were performed with the label-free quantification option selected. Perseus v1.6.2.3. Student's t-test was used for quantitative comparisons, following logarithmic transformation, in order to identify significant differences across the biological replica. Fold changes (FC) were calculated based on the ratio of geometric means of the case versus control samples. Since the PBMC lysates after proteomic analysis contained an extremely limited amount of protein, it was not possible to conduct immunoblots for protein validation in this study.

### 2.6 Statistical analysis

Proteomics data, after logarithmic transformation, were analyzed by 3-way ANOVA and by t-test (Statmodel of Python, version 3.6.4) to determine the effects of treatment: FLX vs. CTL, FLX vs. FO and FO vs. CTL. Differently abundant proteins for each effect were determined at P ≤ 0.05 and absolute FC ±1.5.

## Ethics Statements

The experiment complied with the ARRIVE guidelines and were carried out in accordance with the National Institutes of Health guide for the care and use of laboratory animals (NIH Publications No. 8023, revised 1978).

## CRediT authorship contribution statement

**Gitit Kra:** Methodology, Project administration, Data curation, Visualization, Formal analysis, Validation, Writing – review & editing. **Nataly Nemes-Navon:** Project administration, Data curation, Formal analysis. **Jayasimha Rayalu Daddam:** Data curation, Methodology, Visualization. **Lilya Livshits:** . **Shamay Jacoby:** Data curation, Formal analysis, Methodology. **Yishai Levin:** Data curation, Formal analysis, Methodology, Validation. **Maya Zachut:** Conceptualization, Data curation, Formal analysis, Funding acquisition, Investigation, Methodology, Project administration, Resources, Software, Supervision, Validation, Visualization, Writing – review & editing. **Uzi Moallem:** Conceptualization, Data curation, Formal analysis, Funding acquisition, Investigation, Methodology, Project administration, Resources, Software, Supervision, Validation, Visualization, Writing – review & editing.

## Declaration of Competing Interest

The authors declare that they have no known competing financial interests or personal relationships that could have appeared to influence the work reported in this paper.

## Appendix Supplementary materials

Supplementary Table 1 - Dataset of 3807 identified and quantified proteins obtained by proteomic analysis, as well as statistical analysis of differentially abundant proteins among all groups (*P* ≤ 0.05 and FC ± 1.5) detected in peripheral blood mononuclear in dairy cows supplemented from 257 d of pregnancy with (i) CTL (n = 6) – encapsulated saturated fat, (ii) FLX (n = 6) –encapsulated flaxseed oil providing ALA, or (iii) FO (n = 6) – encapsulated fish oil providing EPA and DHA. Image, application 1

Supplementary Table 2 - The full list of peptides detected in peripheral blood mononuclear in dairy cows supplemented from 257 d of pregnancy with (i) CTL (n = 6) – encapsulated saturated fat, (ii) FLX (n = 6) –encapsulated flaxseed oil providing ALA, or (iii) FO (n = 6) – encapsulated fish oil providing EPA and DHA. Image, application 2

## Footnotes

Supplementary material associated with this article can be found in the online version at doi:10.1016/j.dib.2021.107785.
